# Functional analysis of *Wolbachia* Cid effectors unravels cooperative interactions to target host chromatin during replication

**DOI:** 10.1371/journal.ppat.1011211

**Published:** 2023-03-16

**Authors:** Kévin Terretaz, Béatrice Horard, Mylène Weill, Benjamin Loppin, Frédéric Landmann

**Affiliations:** 1 CRBM, Université de Montpellier, CNRS, Montpellier, France; 2 LBMC, Ecole Normale Supérieure de Lyon, CNRS UMR5239, Université Claude Bernard Lyon 1, Lyon, France; 3 ISEM, Université de Montpellier, CNRS, IRD, Montpellier, France; University of Cambridge, UNITED KINGDOM

## Abstract

*Wolbachia* are common bacteria among terrestrial arthropods. These endosymbionts transmitted through the female germline manipulate their host reproduction through several mechanisms whose most prevalent form called Cytoplasmic Incompatibility -CI- is a conditional sterility syndrome eventually favoring the infected progeny. Upon fertilization, the sperm derived from an infected male is only compatible with an egg harboring a compatible *Wolbachia* strain, this sperm leading otherwise to embryonic death. The *Wolbachia* Cif factors CidA and CidB responsible for CI and its neutralization function as a Toxin-Antitoxin system in the mosquito host *Culex pipiens*. However, the mechanism of CidB toxicity and its neutralization by the CidA antitoxin remain unexplored. Using transfected insect cell lines to perform a structure-function analysis of these effectors, we show that both CidA and CidB are chromatin interactors and CidA anchors CidB to the chromatin in a cell-cycle dependent-manner. In absence of CidA, the CidB toxin localizes to its own chromatin microenvironment and acts by preventing S-phase completion, independently of its deubiquitylase -DUB- domain. Experiments with transgenic *Drosophila* show that CidB DUB domain is required together with CidA during spermatogenesis to stabilize the CidA-CidB complex. Our study defines CidB functional regions and paves the way to elucidate the mechanism of its toxicity.

## Author summary

*Wolbachia* are common Rickettsiales among terrestrial arthropods species and are transmitted vertically through the female germline. These endosymbionts can cause Cytoplasmic Incompatibility -CI-, which is their major form of reproductive manipulation in insects. This sterility syndrome favors the spread of *Wolbachia* in the host population because eggs fertilized by a sperm from an infected male only develop properly when the female is infected with the same or a compatible *Wolbachia* strain. In contrast, if the sperm derived from an infected male fertilizes a non-infected egg or an egg harboring a non-compatible *Wolbachia* strain, CI leads to embryonic death. *Culex pipiens* mosquitos are infected with *Wolbachia w*Pip endosymbionts. *w*Pip Cid effectors causing CI function as a Toxin (CidB) /Antixotin (CidA) system. CidB is loaded without CidA in the maturing sperm to cause CI, while CidA neutralizes CidB during spermatogenesis up to the histone-to-protamine transition and in the compatible *Wolbachia*-infected egg. We explored through mutagenesis approaches how CidB causes cellular toxicity and how it is prevented by CidA. This allowed us to identify CidB and CidA functional domains including chromatin binding domains found in both effectors. CidA transports, stabilizes and neutralizes CidB on the chromatin, preventing the toxin to otherwise block S-phase completion.

## Introduction

*Wolbachia* endosymbionts are Rickettsiales bacteria infecting a majority of terrestrial arthropod species [1,2]. Their outstanding evolutionary success relies in part on powerful strategies of reproduction hijacking of their host to ensure their matriline transmission. The most common mechanism of manipulation, called Cytoplasmic Incompatibility -CI-, is a conditional sterility syndrome [3,4]. CI occurs when a *Wolbachia*-infected male inseminates a female which is either non-infected or harbors another incompatible *Wolbachia* strain. Fertilized eggs resulting from this cross usually arrest at very early stages of development, due to paternal chromosomes segregation defects during the first zygotic division [5]. When the female host is infected with a compatible strain, viable embryo development occurs. Hence, by favoring infected females over uninfected ones, CI is a driving force for *Wolbachia*, allowing the bacteria to spread within its host species. Because of *Wolbachia* ability to interfere with the transmission of numerous arboviruses by *Aedes* mosquito vectors, these endosymbionts are currently used in biocontrol strategies and understanding the mechanisms governing CI can prove crucial in the future [6].

CI involves a pair of effectors collectively known as CI-inducing factors Cifs. They have been classified into different types based on the presence of a deubiquitylation -DUB- domain -called Cids-, nuclease domains only -Cins-, or both -Cinds- [7]. Cid effectors are present in WO prophage region of *Wolbachia* genome as a single operon i.e. in *w*Mel from *Drosophila melanogaster* or as multiple copies i.e. in *w*Pip from the mosquito *Culex pipiens* [8–10].

*In vivo* studies of Cid^**wPip**^ variants with transgenic *D*. *melanogaster* [8,11], transgenic *Anopheles gambiae* mosquitoes [12], as well as *in cellulo* studies with heterologous systems such as the yeast *S*. *cerevisiae* [8,13] and the *D*. *melanogaster* cells S2R+ [11] have led to a Toxin-Antitoxin -TA- model, in which CidA^**wPip**^ is responsible for CidB^**wPip**^ neutralization during spermatogenesis and in compatible crosses, while maternal expression of CidA prevents the egg toxicity of paternally-transmitted CidB. The binding of the CidA^**wPip**^ antitoxin to the CidB^**wPip**^ toxin has been structurally and functionally characterized *in vitro* and revealed three contact regions between Cid^**wPip**^ cognate partners [13,14].

Our recent study of Cid^**wPip**^ effectors spatial and temporal dynamics during gametogenesis and fertilization shed a new light on their functions and mechanisms of action in CI and its rescue [11]. These analyses of *D*. *melanogaster* transgenic lines showed Cid^**wPip**^ effectors to be present together in the male germline. The toxin remains presumably bound to its antitoxin *in vivo* and hence neutralized during male germ cell proliferation and meiosis until spermiogenesis and the histone-to-protamine transition. At this crucial step, CidA^**wPip**^ gets evicted from maturing sperm nuclei while in contrast the toxin CidB^**wPip**^ remains. The persistence of endogenous cidB in mature spermatids was confirmed in wPip infected *C*. *pipiens* [10]. In the fertilized egg, the paternally-transmitted toxin colocalizes with DNA replication stress markers on the paternal chromosomes, suggesting that S-phase may be directly affected by CidB^**wPip**^. Importantly, we showed that catalytically dead DUB mutants of CidB^**wPip**^ do not prevent neither cellular toxicity *in vitro* nor CI induction *in vivo*, while the DUB domain catalytic activity was originally considered the direct cause of CI [8].

The growing knowledge of Cid^**wPip**^ effectors have set a solid ground to further explore the mechanisms of neutralization of CidB^**wPip**^ by CidA^**wPip**^ and how the toxin acts otherwise to induce cellular defects. Specifically, what is the cellular mechanism targeted by CidB^**wPip**^, which domains are involved and how does CidA^**wPip**^ binding prevent CidB^**wPip**^ toxicity are open questions. We addressed here some of them by performing a structure-function analysis of a pair of Cid^**wPip**^ effectors and investigated the ability of CidB to affect DNA replication.

We first performed a thorough mutagenesis using the *D*. *melanogaster* S2R+ cell heterologous system. Such *in cellulo* strategy offers the possibility to quickly test a significant number of mutants before turning to a transgenic flies-based *in vivo* approach. S2R+ cells allow to dissect the mechanisms of CidB toxicity and its neutralization by the CidA antitoxin, with additional information regarding the subcellular localization of these events thanks to wild-type and mutant fluorescent Cid reporters. Moreover, cellular transfections make possible the expression of Cid effectors separately to establish their impact on cell cycle and to uncover features otherwise masked during their association as heterodimers, since transgenic *D*. *melanogaster* lines expressing CidB only cannot be established [8]. The distribution and the toxicity of a selection of relevant Cid mutants was subsequently evaluated *in vivo* thanks to the establishment of *D*. *melanogaster* transgenic lines.

This experimental approach allowed us to demonstrate that both CidA and CidB have the capacity to interact with chromatin, although CidB preferentially associates with CidA rather than with chromatin. We show that CidA binds CidB to neutralize it independently of their subcellular localization. Cytological observations of *D*. *melanogaster* transgenic testes indicate that the C-terminus part of CidB containing the DUB domain contributes together with CidA to protect CidA-CidB complexes from degradation mechanisms during spermatogenesis, eventually allowing the CidB toxin to be properly loaded in maturing sperm. Finally, cellular analyses in transfected S2R+ cells indicate that CidB impacts DNA replication completion independently of the DUB domain. This structure-function analysis of CidB sheds light on its functional regions and their respective roles in driving toxicity, bringing essential insights toward the discovery of CidB targets in CI.

## Results

### CidA and CidB effectors dynamically colocalize during the cell cycle

**Fig 1:**
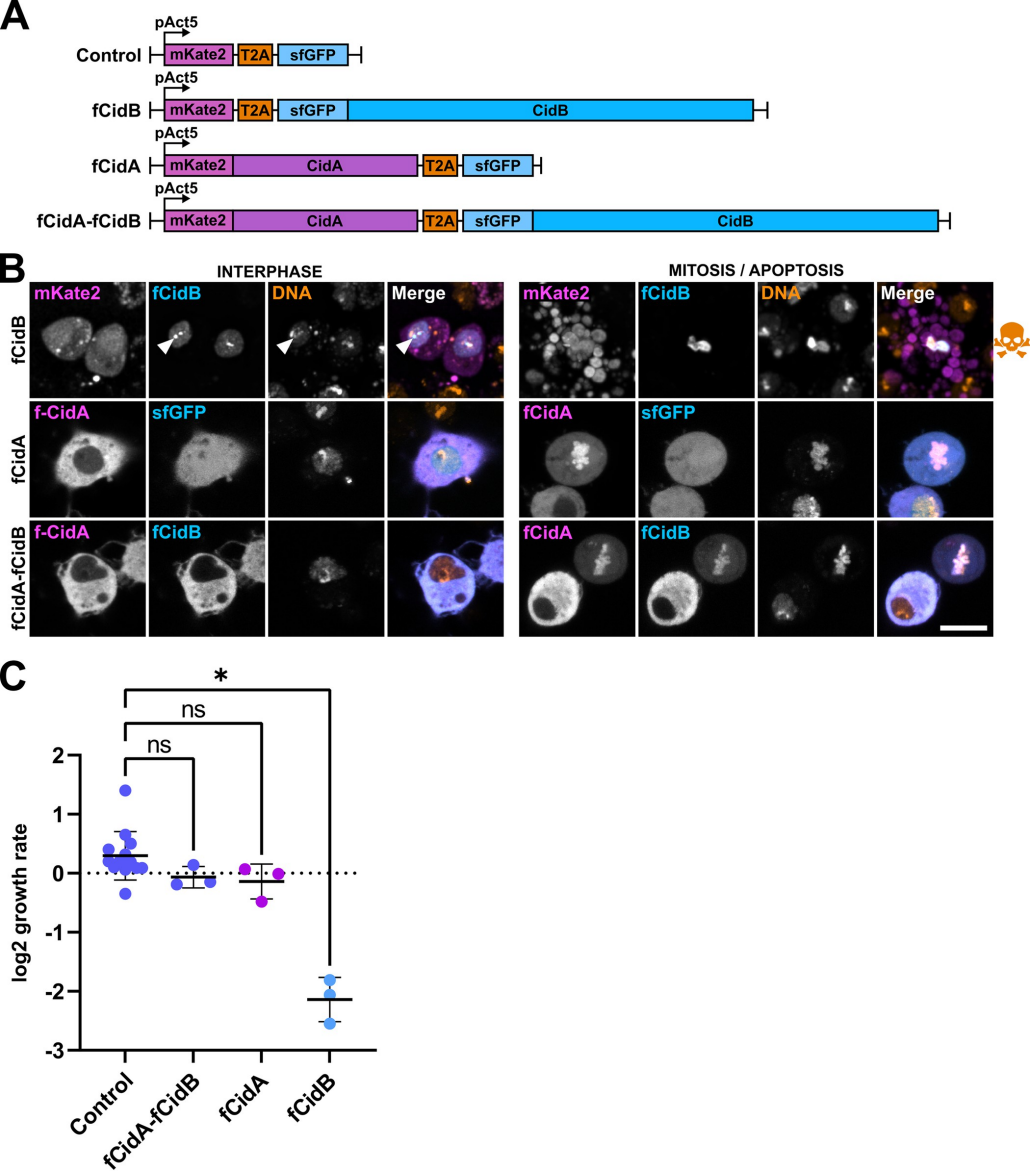
CidA_IV(δ/1) and CidB_IV(a/2) function as a Toxin-Antidote system in S2R+ cells. (A) Schematic drawing of the constructs used to express fCidA and fCidB and in FACS analyses. Fluorescent effectors expressed alone are co-expressed with an additional free fluorochrome, mKate2 or superfolder GFP thanks to the T2A self-cleaving peptide, all under control of the constitutive promoter of *D*. *melanogaster* Actine 5C. All Cid mutants expressed as fluorescent proteins presented in this study are derived from these constructs. (B) Confocal images of transfected cells expressing fCidA or the free mkate2 in magenta, fCidB or the sfGFP in cyan, and the DNA stained with Hoechst in yellow. The left panel shows cells in interphase, the right panel shows either cells in mitosis or during apoptosis indicated by a skull and crossbones symbol. In interphase, arrowheads point toward heterochromatin-associated perinucleolar CidB foci. Scale bar = 10 μm. For each condition, n>100 transfected cells observed. (C) FACS analyses of transfected S2R+ cells growth expressed as a ratio of fluorescent cells between day 4 and day 2 post-transfection (see Methods). The Y axis represents a log2 fold change in cell growth. Values close to 0 reflect cell growth comparable to non-transfected cells while negative values are obtained when transfected cells die or stop growing. Each dot represents a biological replicate. Middle bar is mean, error bars are SD. The * indicates a p < 0.01 with a one-way ANOVA followed by multiple comparisons.

The *Wolbachia w*Pip strains from *C*. *pipiens* recently diverged into five distinct phylogenetic groups (wPipI to wPipV) [15]. They all contain a large diversity of *cidA* and *cidB* genes, amplified and diversified within each *w*Pip genome [9]. While previous analyses have focused on a pair of *cidA* and *cidB* genes from wPip group III [8,14], we selected here another variant, CidB_IV(a/2), because this CidB is largely associated with CI induction in wPip group IV strains. CidA_IV(δ/1) was used as its cognate partner because its presence in every strains harboring CidB_IV(a/2) strongly suggested a rescuing ability [9] (S1 Fig). We transfected *D*. *melanogaster* S2R+ cells with constructs allowing the expression of these CidA and CidB variants either as single effectors or in combination thanks to a self-cleaving peptide T2A mimicking the operon organization of cognate partners in *w*Pip [8] (Fig 1A). CidA and CidB were expressed as fluorescent protein fusions mKate2::CidA and sfGFP::CidB named hereafter fCidA and fCidB, and we analyzed their subcellular localization and their impact on cell growth by confocal microscopy as well as FACS analyses (Fig 1). Similarly to what we previously reported with a pair of Cid variants from group III, fCidB localizes throughout the nucleus and its expression prevents mitosis to eventually lead to apoptosis (Fig 1B), even at very low levels of expression, as indicated by the fluorescence intensity of the sfGFP reporter (S1 Movie). FACS-based cell growth assays indicate that the population of cells expressing fCidB dramatically collapses from day 2 to day 4 post-transfection, despite transfection events occurring continuously during the experiment (Fig 1C). Analyses of time-lapse experiments confirm the robust toxicity of fCidB, that prevents any cell division in transfected cells to instead induce 92% apoptosis over a period of 48 hours (n = 429, S2 Fig). We found fCidB enriched in heterochromatin regions located around the nucleolus in *D*. *melanogaster* S2 cells [16] and brightly stained by Hoechst DNA dye, suggesting that CidB could preferentially interact with specific chromatin environments (Fig 1B and S2 Movie, arrowheads). When expressed alone, the vast majority of fCidA is cytoplasmic in interphase, and after Nuclear Envelope Breakdown -NEB-, fCidA decorates the condensed chromosomes (Fig 1B). After nuclear envelope assembly, fCidA largely returns to the cytoplasm and does not affect cell growth (Fig 1C). Neither mitotic events (44%) nor apoptotic events (4%) scored in transfected cells over 48 hours in time-lapse experiments (n = 458) appear different from the control (40%, 6% respectively, n = 310) (S2 Fig). When effectors are co-expressed, fCidA prevents fCidB nuclear import during interphase and both effectors decorate the condensed chromatin during mitosis. fCidB always dynamically colocalizes with fCidA during cell cycle (Fig 1B). Their co-expression does not affect the cell growth rate compared to the control (Fig 1B and 1C). In 48hr-long time-lapse experiments, fCidA-fCidB expressing cells show mitotic (37%) and apoptotic events (6%, n = 665) comparable to the control (S2 Fig). Together, these experiments suggest that these Cid variants from group IV function as a Toxin-Antitoxin system.

### The binding of CidB to CidA is sufficient to prevent cell division defects independently of the subcellular localization of the complex

**Fig 2:**
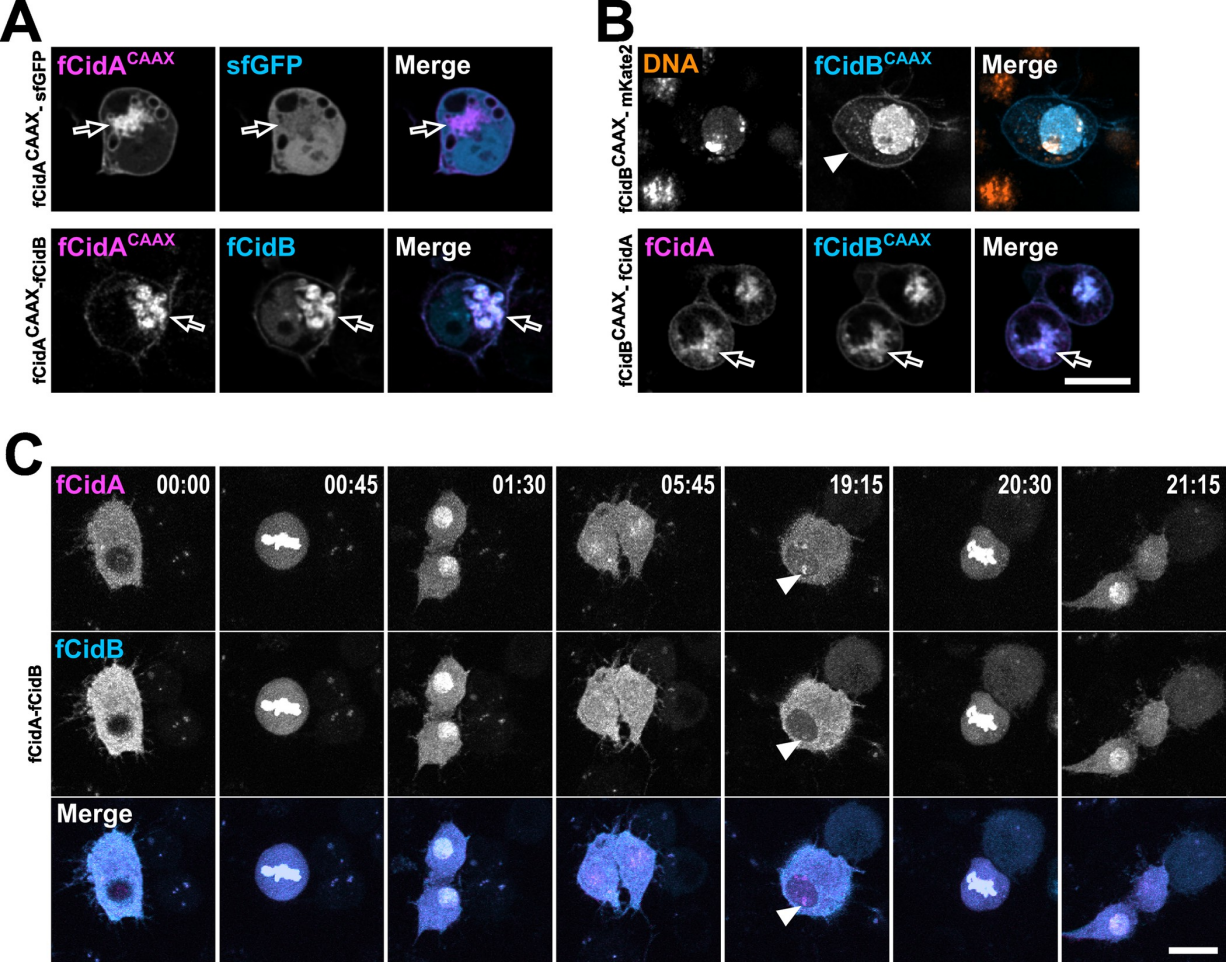
*In cellulo* physical interaction of CidA with CifB is sufficient to neutralize CidB. (A) Confocal images of S2R+ cells expressing fCidA^**CAAX**^ together with fCidB or alone. FCidA^**CAAX**^ decorates the cytoplasmic side of cellular membranes and relocalizes CidB possibly to endoplasmic reticulum (arrow) and vesicular membranes as well as plasma membrane unlike the free GFP that remains cytoplasmic and nuclear. (B) S2R+ cells expressing fCidB^**CAAX**^ alone or together with fCidA. The arrowhead points to fCidB^**CAAX**^ at the cell membrane, the arrow points to fCidA and fCidB^**CAAX**^ at membranes. (C) A 24-hr time lapse experiment showing the dynamics of fCidA-fCidB across two cell divisions. After mitosis the Cid effectors are slowly exported out of the nuclei (from 1:30 to 19:15 hrs) although a fraction remains in the nucleus and also associates with the chromatin -arrowheads- without preventing next mitosis (20:30). Scale bars = 10 μm.

To confirm that a direct binding of CidA to CidB occurs during interphase *in cellulo*, we added a CAAX prenylation signal sequence to force CidA or CidB to localize at the cytoplasmic surface of cellular membranes and endoplasmic reticulum [17] (Fig 2A and 2B). Live imaging shows that when co-expressed with fCidB, fCidA^**CAAX**^ anchors its cognate partner at membrane compartments without triggering cell death, demonstrating that a physical interaction is responsible for fCidB neutralization and nuclear import inhibition (Fig 2A). In order to test the cellular toxicity of fCidB when kept away from the nucleus, we next added a CAAX sequence to this protein. Contrary to our hypothesis, most of fCidB^**CAAX**^ remains nuclear, while only a small fraction decorates cell membranes (Fig 2B arrowhead). Nonetheless fCidB^**CAAX**^ fully colocalizes with fCidA to membrane compartments when co-expressed (Fig 2B arrow). These reciprocal prenylation experiments of Cid effectors demonstrate the direct binding of CidA to CidB *in cellulo*. In addition, when fCidA and fCidB are co-expressed, time-lapse experiments reveal incomplete nuclear export after the first mitosis occurring post-transfection, resulting in a nuclear localization of fCidA-fCidB complexes during interphase that does not affect cell division (Fig 2C and S3 Movie).

### The DUB domain is dispensable to CidB toxicity that relies on a chromatin interaction

**Fig 3:**
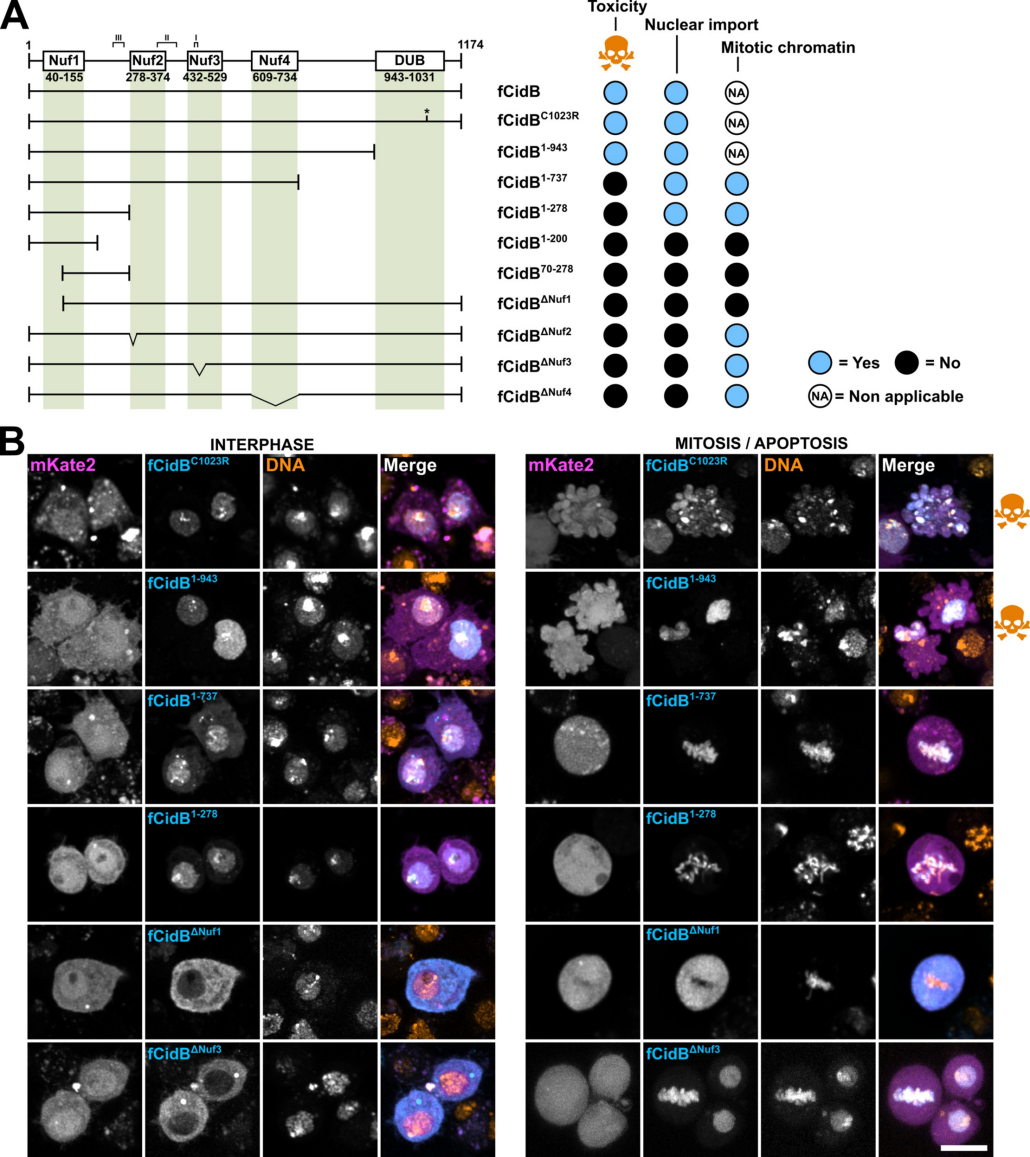
Structure-function analysis of CidB. (A) Schematic representation of CidB with its previously identified domains, together with the various deletion mutants tested and associated phenotypes. When CidB or its mutants are toxic and prevent mitosis, the localization to the mitotic chromatin is non applicable. (B) Confocal images of S2R+ cells transfected with fCidB mutants showing the subcellular localization in interphase and its outcome illustrated either by mitotic figures or apoptotic events -blebbing cells with fragmented DNA, skull and crossbones symbol-. CidB mutants are in cyan, free mkate2 in magenta and DNA -Hoechst- in yellow. Scale bar = 10 μm.

We performed a structure-function analysis of CidB to gain insights into the domains responsible for its localization and cellular toxicity. We created a series of fCidB mutants and observed transfected cells by confocal microscopy to determine mitotic or apoptotic events over a period of 2 days post-transfection, a duration in which cells can undergo several rounds of division (Figs 3 and S2). By protein sequence comparison with CidB^*w*Pip^ used in previous studies [8,14] we mapped and named the four catalytically inactive Nuclease fold domains hereafter Nuf1,2,3 and 4, spanning residues 40–155, 278–374, 432–529 and 609–734 respectively in CidB_IV(a/2) as well as the ULP-like domain endowed with a deubiquitylase activity -DUB- spanning residues 943 to 1031. We also highlighted the three CidB_IV(a/2) regions of physical interface with CidA_IV(δ/1), spanning residues 450–460, 350–401, and 231–261 for interfaces I, II and III respectively (Figs 3 and S1).

Although this toxin was originally considered to directly induce CI thanks to the DUB domain [8], our previous analyses of a catalytic inactive C1025A CidB mutant suggest that the DUB domain is dispensable to induce toxicity [11]. To block the DUB activity and reduce the affinity for ubiquitin of the CidB variant chosen for this study, we substituted the cysteine of the DUB catalytic triad by an arginine [18] in fCidB^**C1023R**^. This mutant confirmed that the DUB activity is dispensable to induce toxicity (Figs 3A, 3B and S2 Fig).

Next, in order to identify the minimal CidB fragment inducing toxicity, we sequentially shortened the protein from its C-terminus part and created the three following deletion mutants. fCidB^**1-943**^ deletes a C-terminus part encompassing the full DUB domain, fCidB^**1-737**^ stops after Nuf4 and fCidB^**1-278**^ before Nuf2 (Fig 3A). We noticed that these three fCidB mutants are inherently less stable than any others. fCidB^**1-943**^ is imported to the nucleus but its GFP level decreases in time lapse experiments in a majority of cells to become faint perinucleolar foci only (S3 Fig). Remarkably, this mutant deleted of the DUB domain still induced 34% apoptotic events in time lapse experiments (n = 623, Figs S2 and 3B). Together, this dataset suggests that the deleted domain in fCidB^**1-943**^ confers stability to CidB and confirms that the DUB domain is dispensable to CidB toxicity *in cellulo*.

fCidB^**1-737**^ and fCidB^**1-278**^ are also imported to the nucleus, but in contrast to fCidB^**1-943**^ do not induce apoptosis (Figs 3B and S2). Instead, they associate with the condensed chromatin in mitosis. Of note, fCidB^**1-737**^ forms cytoplasmic aggregates in addition to its nuclear localization (S3 Fig). To explain the phenotypic differences between fCidB^**1-737**^ and fCidB^**1-943**^ both deleting the DUB domain, we hypothesize that the interdomain region (737–943) participates either to the proper folding of CidB and/or to interactions with a host target essential to trigger cell death.

Next, we tried to further narrow down the chromatin associated domain -CAD- contained in the loss-of-function mutant fCidB^**1-278**^. Our failed attempts to maintain a chromatin binding, i.e. with fCidB^**1-200**^ and fCidB^**70-278**^, suggest that the CAD is larger than Nuf1 and encompassed in the 278 residue-long N-terminus region (Fig 3A and 3B). In addition, we created a truncation of the first hundred amino acids in fCidB^**ΔNuf1**^. This mutant prevents nuclear import and inactivates the toxin, which becomes unable to bind to the chromatin after NEB (Fig 3A and 3B). Together these experiments indicate that CidB is able to bind to the chromatin on its own thanks to a CAD necessary but not sufficient to confer toxicity. Moreover, although the DUB domain seems to participate to the protein stability, it is not essential to prevent mitosis and trigger apoptosis in *D*. *melanogaster* S2R+ cells.

Finally, we disrupted the three other pseudonuclease domains (Nuf2 to 4) that are not necessary to the chromatin binding of CidB. Nuf2 was deprived of its first alpha helix (residues 278 to 293) in fCidB^**ΔNuf2**^, Nuf3 was deleted of a region whose polymorphism has been previously correlated to compatibility between cognate partners (residues 451 to 481) in fCidB^**ΔNuf3**^ [9] and the Nuf4 domain was entirely deleted in fCidB^**ΔNuf4**^. These three deletion mutants of CidB lead to a same phenotype. They surprisingly remain in the cytoplasm during interphase up to the first mitosis, despite the presence of the first 278 residues sufficient to trigger nuclear import (fCidB^**1-278**^). Nonetheless they do bind as expected onto the chromatin after NEB without preventing mitosis. Of note, they next mostly remain in the nucleus where their localization is similar to wild-type CidB in interphase.

Because deletions in the 278–943 region prevent the 1–278 region-driven nuclear import during interphase, we hypothesize that induced conformational changes prevent this import. This structure-function analysis of CidB also reveals that any deletions in the CAD or the 278–943 region abrogate CidB cellular toxicity even in presence of the DUB domain.

### A CidA functional analysis reveals a chromatin binding domain

**Fig 4:**
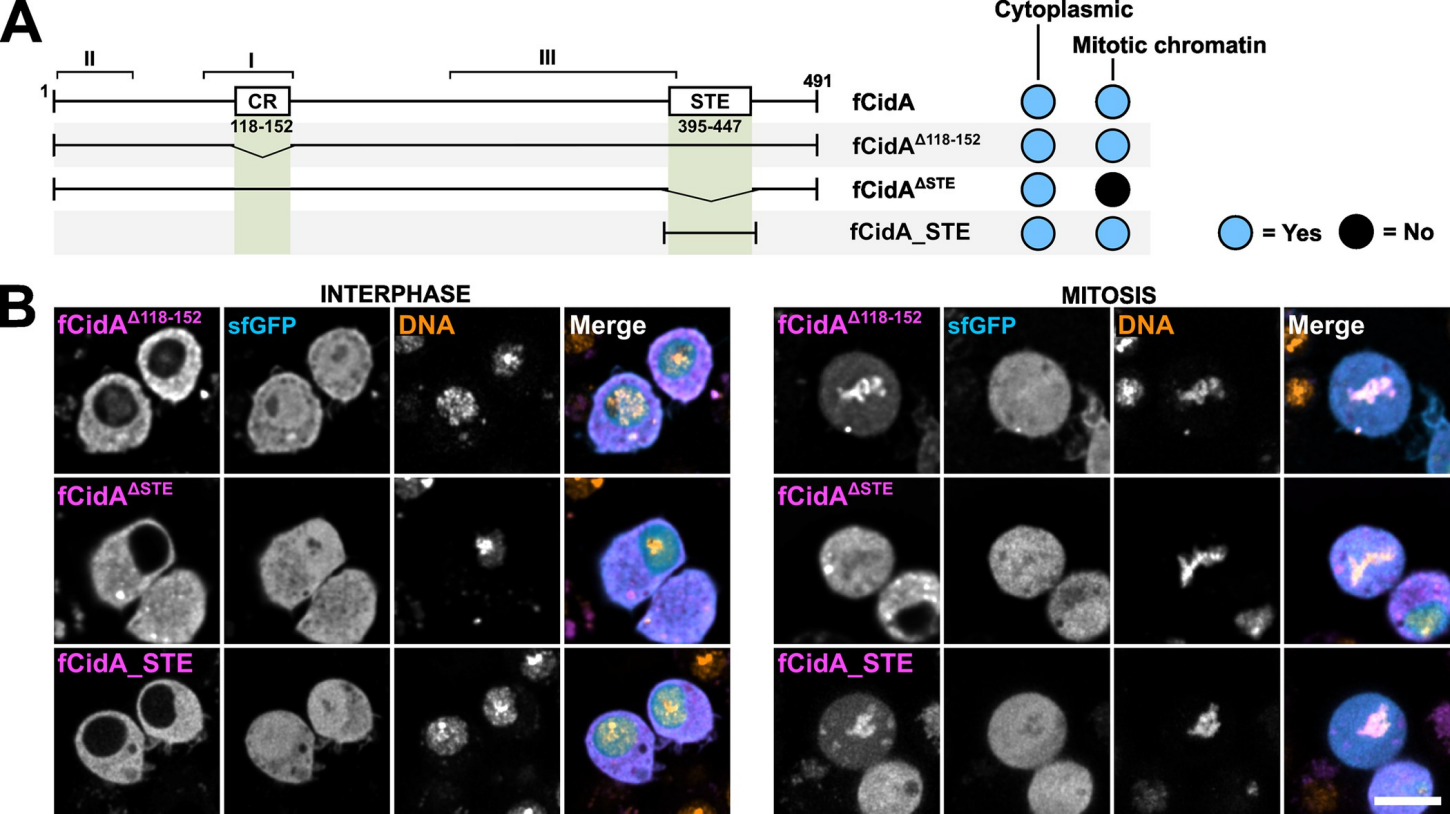
CidA STE is a chromatin binding domain. (A) Schematic view of CidA and its putative domains tested with deletion mutants and their subcellular localization in interphase (i.e. cytoplasmic) and in mitosis (i.e. chromatin) in S2R+ cells. (B) Confocal images of cells expressing the CidA mutants presented in (A). CidA mutants are in magenta, sfGFP in cyan and DNA -Hoechst- in yellow. Scale bar = 10 μm.

We established that CidA associates with the chromatin, in addition to its previously characterized binding to CidB for which the regions of interaction were mapped in detail by structural studies [13,14]. To identify domains governing CidA localization and dynamics, we first deleted in fCidA^**Δ118–152**^ a Correlated Region -CR- whose polymorphism is correlated to compatibility between cognate partners [9], that largely affects Interface I [14] (Figs 4A and S1). This mutant displays a similar dynamic localization to that of wild-type fCidA expressed alone (Figs 1 and 4B). Next, we deleted a domain identified as a putative sterile-like transcription factor domain STE-like (residues 395 to 447) [19], called hereafter “STE” for the sake of clarity. fCidA^**ΔSTE**^ mutant is unable to bind to the condensed chromatin during mitosis (Fig 4A and 4B). Conversely, the expression of a fragment composed of 52 residues (391–453) encompassing STE in fCidA_STE decorates the chromatin in mitosis, and constitutes *bona fide* the CidA CAD.

### Interactions between wild-type and mutant Cid effectors suggest differential selective affinities between cognate partners and their chromatin targets

In addition to physically associate, CidA and CidB are also able to interact with their own, yet unidentified, chromatin targets. What are the mechanisms governing the preference for the cognate partner over host targets? When both effectors are localized onto the chromatin and how is this association scaffolded? To shed light on these questions, we characterized the protein domains necessary for the binding of the Cid effectors together and to their targets. To this end, we combined wild-type and mutant versions of CidA and CidB in co-expression studies to take advantage of their subcellular localizations informing on their interacting partners.

**Fig 5:**
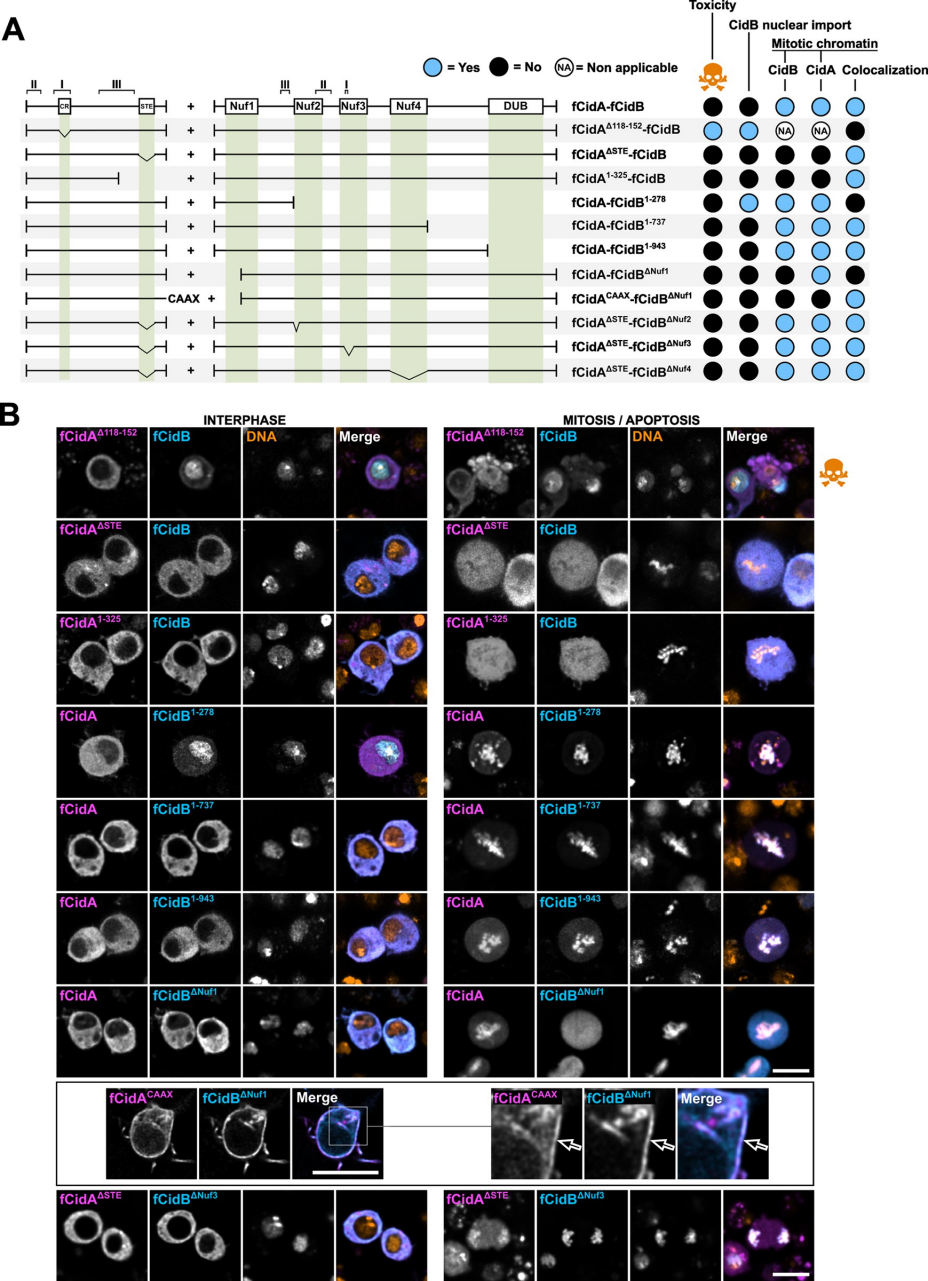
Phenotypes resulting from CidA-CidB mutants co-expression *in cellulo*. (A) Schematic drawing of fCidA and fCidB wild-type and mutant combinations co-expressed with the T2A self-cleaving peptide under the pAc5 promoter. The resulting phenotypes presented on the right include the toxicity (i.e. no mitosis and apoptotic events observed), the CidB nuclear import during interphase, and the CidA and CidB mitotic localizations (i.e. on the chromatin). (B) Confocal images of cells expressing the constructs presented in (A) in interphase and mitosis or apoptosis -skull and crossbones symbol-. Images of CidB^**ΔNuf1**^- CidA^**CAAX**^ co-expression are presented separately in a box with an enlargement on the right. CidA in magenta, CidB in cyan and DNA -Hoechst- in yellow. Scale bar = 10 μm.

First, we tested the ability of CidA deletion mutants to bind to CidB and thus prevent its toxic effect in the cell (Figs 5A, 5B and S2). Expressed alone, fCidA^**Δ118–152**^ has a dynamic localization undistinguishable from that of fCidA (Fig 4). Interestingly, however, fCidA^**Δ118–152**^ does not bind CidB, which appears nuclear in interphasic cells and induces apoptosis. This loss of interaction reveals a crucial role for CidA interface I in CidB binding and neutralization (Fig 5). When the mutant fCidA^**ΔSTE**^ unable to bind to the chromatin is co-expressed with fCidB, it sequesters the toxin in the cytoplasm in interphase and importantly prevents fCidB localization to condensed chromatin in mitosis. This suggests that when interfaces I, II and III are all present in both partners, fCidB shows an affinity greater for fCidA^**ΔSTE**^ than for the chromatin. This experiment also indicates that the CidA STE domain is not required to neutralize CidB and that a wild-type CidA-CidB complex very likely localizes to the chromatin thanks to the CidA STE domain. Next, we searched for the shortest fCidA fragment able to neutralize fCidB by co-expressing a series of CidA deletion mutants and we identified fCidA^**1-325**^ as the shortest stable deletion mutant (Fig 5A and 5B). This mutant disrupts CidA interface III spanning from residue 256 to 400. It suggests that an intact interface III in CidA is not necessary to allow a neutralizing binding to CidB. This is in agreement with a mutagenesis in CidA^**wMel**^ targeting the same region, producing point mutants still able to rescue CI [20]. Further attempts to test the capacity of CidA mutants to neutralize fCidB (fCidA^**1-200**^ deleting full interface III or fCidA^**Δ1–100**^ deleting interface II) were unsuccessful since these mutants were not stably expressed, resulting in low fluorescence levels or in the presence of cytoplasmic aggregates (S4 Fig).

Similarly, we sought to determine which CidB regions are essential to the binding with CidA by co-expressing a series of mutants with fCidA. We started with mutants deleting either large regions covering several domains or the DUB domain only, in fCidB^**1-278**^, fCidB^**1-737**^ and fCidB^**1-943**^ (Fig 5A and 5B). fCidB^**1-278**^ and fCidA occupy different sub-cellular compartments in interphase -the nucleus and the cytoplasm respectively- and therefore do not interact. Hence, the interface III remaining in this CidB mutant is not sufficient to allow a physical interaction with CidA. In contrast, both fCidB^**1-737**^ and fCidB^**1-943**^ physically interact with their CidA cognate partner and remain cytoplasmic in interphase, which is in agreement with the crystal structure of the CidA-CidB complex obtained without the DUB domain [14]. Expression patterns of CidB mutants in single transfection versus in co-expression experiments with fCidA reveal a stabilizing role of the antidote. In presence of fCidA, we observed that fCidB^**1-943**^ fluorescence level is maintained, and fCidB^**1-737**^ stops forming cytoplasmic aggregates (S3 Fig).

In order to evaluate the role of the Nuf domains in CidB’s interactions with CidA and with the chromatin, deletion mutants disrupting a domain’s function were co-expressed with wild-type CidA. When fCidA is co-expressed with the loss-of-function mutant fCidB^**ΔNuf1**^ **(**deleting the first 100 N-terminus residues and disrupting both CidB Nuf1 and CAD), only fCidA decorates the condensed chromatin after NEB. Although the three interfaces are present in this mutant, this intriguingly suggests that fCidA has nonetheless a greater affinity for its chromatin target than for fCidB^**ΔNuf1**^. Therefore, to test a potential interference created by the chromatin in this CidA-CidB mutant interaction, we co-expressed a membrane-bound fCidA^**CAAX**^ together with fCidB^**ΔNuf1**^. In absence of a competing chromatin target of CidA sequestered in the cytoplasm, the interaction between the cognate partners is maintained, as observed by their colocalization at the membrane (Fig 5A and 5B). Taken together, these two experiments suggest that the interaction of CidA with a CidB deprived of its first 100 N-terminus residues is weaker than CidA interaction with its chromatin target. Although this N-terminus region of CidB is outside the three interfaces of contact with CidA, its deletion could likely induce a conformational change weakening contact sites between cognate partners, making CidA unable to simultaneously interact with its chromatin target and a fCidB^**ΔNuf1**^ mutant.

We established that the deletions created in fCidB^**ΔNuf2**^, fCidB^**ΔNuf3**^ and fCidB^**ΔNuf4**^ lead to a common phenotype. When expressed alone, all these three loss-of-function CidB mutants remain cytoplasmic in interphase and bind to the condensed chromatin after NEB (Fig 3). Because this dynamic localization is identical to that of wild type CidA, a co-expression cannot inform on potential interactions. We chose instead to co-express them all with fCidA^**ΔSTE**^, which prevents the binding of full length CidB to mitotic chromosomes. As a consequence the fCidA^**ΔSTE**^-fCidB complex always remains cytoplasmic even in mitosis. Surprisingly, these three CidB mutants are not sequestered away from the condensed chromatin by fCidA^**ΔSTE**^, but repatriate fCidA^**ΔSTE**^ to the chromatin after NEB (i.e. fCidB^**ΔNuf3**^, Fig 5A and 5B). We postulate that the interaction of these CidB mutants with CidA^**ΔSTE**^ is suboptimal because of the reduced contact sites between cognate partners. The remaining interaction sites may be sufficient to allow a physical binding, but are unable to prevent CidB to interact with chromatin, resulting in an alternative scaffold in which these CidB mutants become linkers between the chromatin and CidA^**ΔSTE**^. These co-expression studies of fCidA^**ΔSTE**^ with CidB mutants deleted in Nuf domains suggest that CidB interface II and III are sufficient to allow binding to CidA. Because fCidB^**ΔNuf2**^ as well as fCidB^**ΔNuf4**^ do not directly affect interfaces, these two mutants are likely to induce conformational changes reducing contact sites between partners.

In summary, this set of experiments indicates a preference of CidB for CidA *in cellulo* over its chromatin targets. Hence, the CidA-CidB heterodimer very likely interacts with the chromatin through the CidA STE-like transcription factor domain. The interaction of CidA with CidB prevents CidB nuclear import, but most importantly its own direct chromatin binding and toxicity. Interfaces I-II of CidA, and II-III of CidB are necessary and sufficient for a neutralization of CidB by CidA.

### Both CidA and the CidB DUB domain protect CidA/CidB complexes from degradation during spermatogenesis

**Fig 6:**
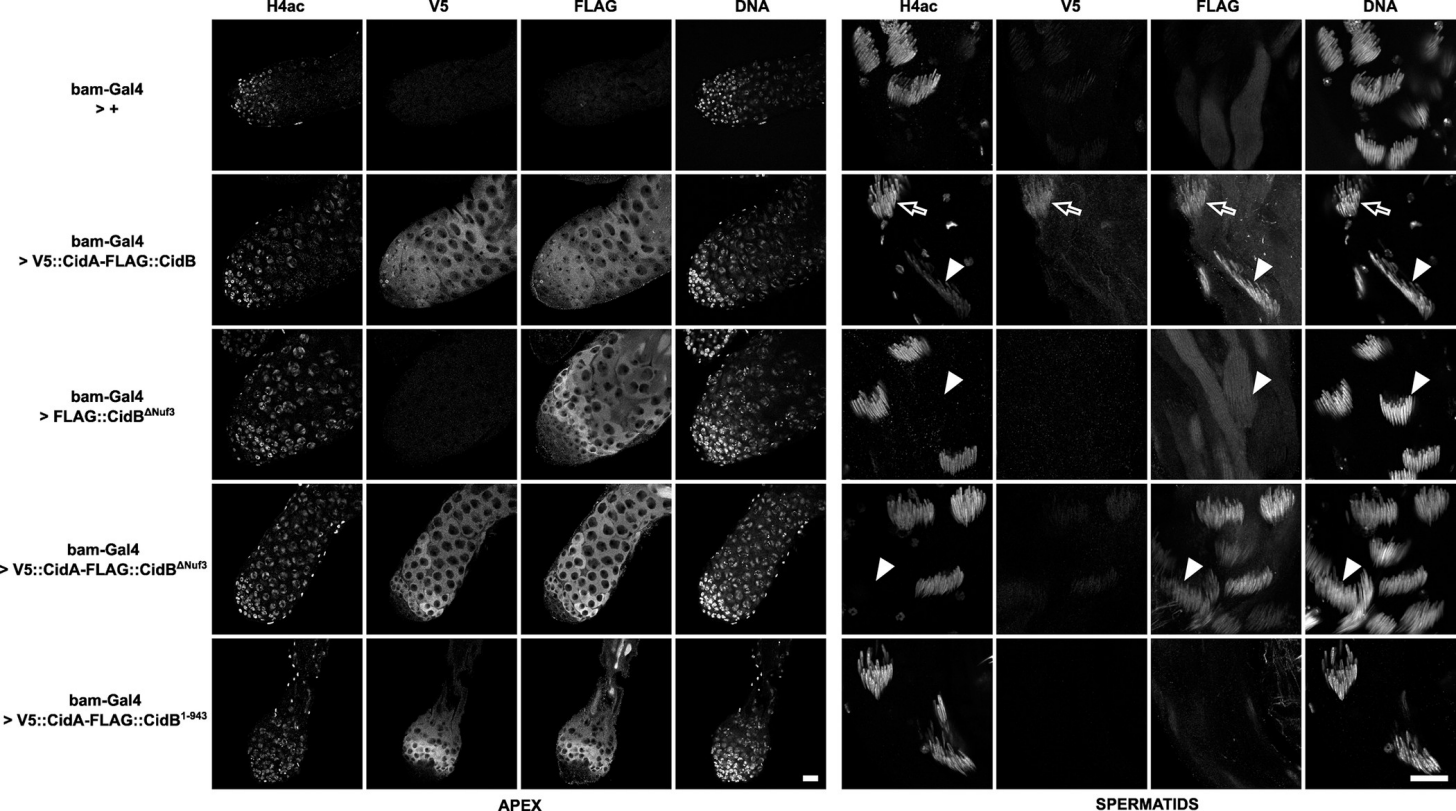
Spatial-temporal localizations of Cid effectors in transgenic *D*. *melanogaster* testes. Confocal images of *D*. *melanogaster* testes of the indicated genotypes. Apical region with proliferative germ cells is shown on the left and canoe stage spermatids on the right. The immunofluorescence stainings reveal from left to right for each zone of the testis the acetylated histone 4 -H4ac- lost during the histone-to-protamine transition, V5::CidA, Flag::CidB, and DNA stained with DAPI. Arrows indicate cysts positive for H4ac before the histone-to-protamine transition while arrowheads point toward post-transition cysts negative for H4ac. The first horizontal row is a negative control testis which only expresses *bam*-Gal4. The testis shown in the second row expresses wild-type tCidA and tCidB. Representative spermatid cysts still positive for histones (H4ac) are indicated with an arrow. Arrowheads show cysts after the histone-to-protamine transition. Scale bars = 20 μm.

To explore additional roles for the CidA antidote *in vivo* and to clarify the function of the CidB DUB domain during spermatogenesis, we created *D*. *melanogaster* transgenic lines expressing tagged versions of wild-type and mutant *Wolbachia* effectors under the control of the inducible UAS/GAL4 system. Here we used the CidA and CidB variants from group III as previously [11]. All transgenes were designed from a basic UAS-V5::CidA-T2A-FLAG::CidB scaffold, hereafter UAS-tCidA-tCidB [8,11]. tCidB was expressed alone or in combination with tCidA using the T2A self-cleaving peptide, thanks to the germline-specific *bam*-Gal4 driver which is expressed in proliferating spermatogonia. Hence the products of these transgenes detected beyond the mitotic proliferation stage are stably present during meiosis and the differentiation of spermatids (spermiogenesis). As previously reported [11], effector proteins expressed in the testes of control *bam-Gal4 > UAS-tCidA-tCidB* males were detected in spermatogonia, spermatocytes and spermatids up to the histone-to-protamine transition. Following histone removal, only tCidB was retained in the nuclei of canoe stage spermatids (Fig 6). Because wild-type tCidB cannot be expressed without tCidA due to its toxicity [8], comparison of its spatial-temporal dynamics with and without tCidA is not possible. To circumvent this limitation, we chose to express the loss-of-function mutant tCidB^**ΔNuf3**^ alone and with tCidA. In *bam-Gal4 > UAS-tCidB*^***ΔNuf3***^ testes, this CidB mutant is detected in spermatogonia and early spermatocytes. In post-meiotic spermatids however, the protein becomes undetectable. Strikingly the co-expression with tCidA fully restores tCidB^**ΔNuf3**^ expression profiles to levels similar to that of the wild-type control. Despite an accumulation of the toxin in maturing spermatid nuclei, these males were fully fertile (S1 Table), thus confirming that the Nuf3 domain of CidB is critical for CI. In addition, we observed that the temporal dynamics of CidA disappearance from the germline that normally coincides with the histone-to-protamine transition occurs much earlier when co-expressed with tCidB^**ΔNuf3**^, typically in round spermatids shortly after meiosis. We hypothesize that the interface reduction between cognate partners weakens the interaction and accelerates tCidA removal during early spermiogenesis. We previously reported that a CidB^***C1025A***^ DUB catalytic mutant, still able to bind to one ubiquitin, whose level was robustly reduced in spermatids, could still induce CI, suggested for the DUB a role of localization or stabilization of CidB in post-meiotic germ cells [11]. Here we analyzed the expression of a DUB domain deletion mutant. Upon expression of a *bam-Gal4 > UAS-tCidA-tCidB*^***1-943***^ transgene, both CidA and CidB are detectable in early, *bam*-Gal4 expressing male germ cells, but become only barely detectable above background noise in spermatids. These males were found to be fully fertile (S1 Table). Hence, the debubiquitylation activity of CidB is indirectly important to promote CI through effectors stability and this could explain why point mutations in the DUB domain of *Wolbachia* from *Drosophila sp*. CidB orthologs affect CI strength [21]. Taken together, this dataset indicates that both CidA and CidB DUB domain contribute to protect the CidA/CidB complex from degradation.

### CidB perturbs the S-phase progression

**Fig 7:**
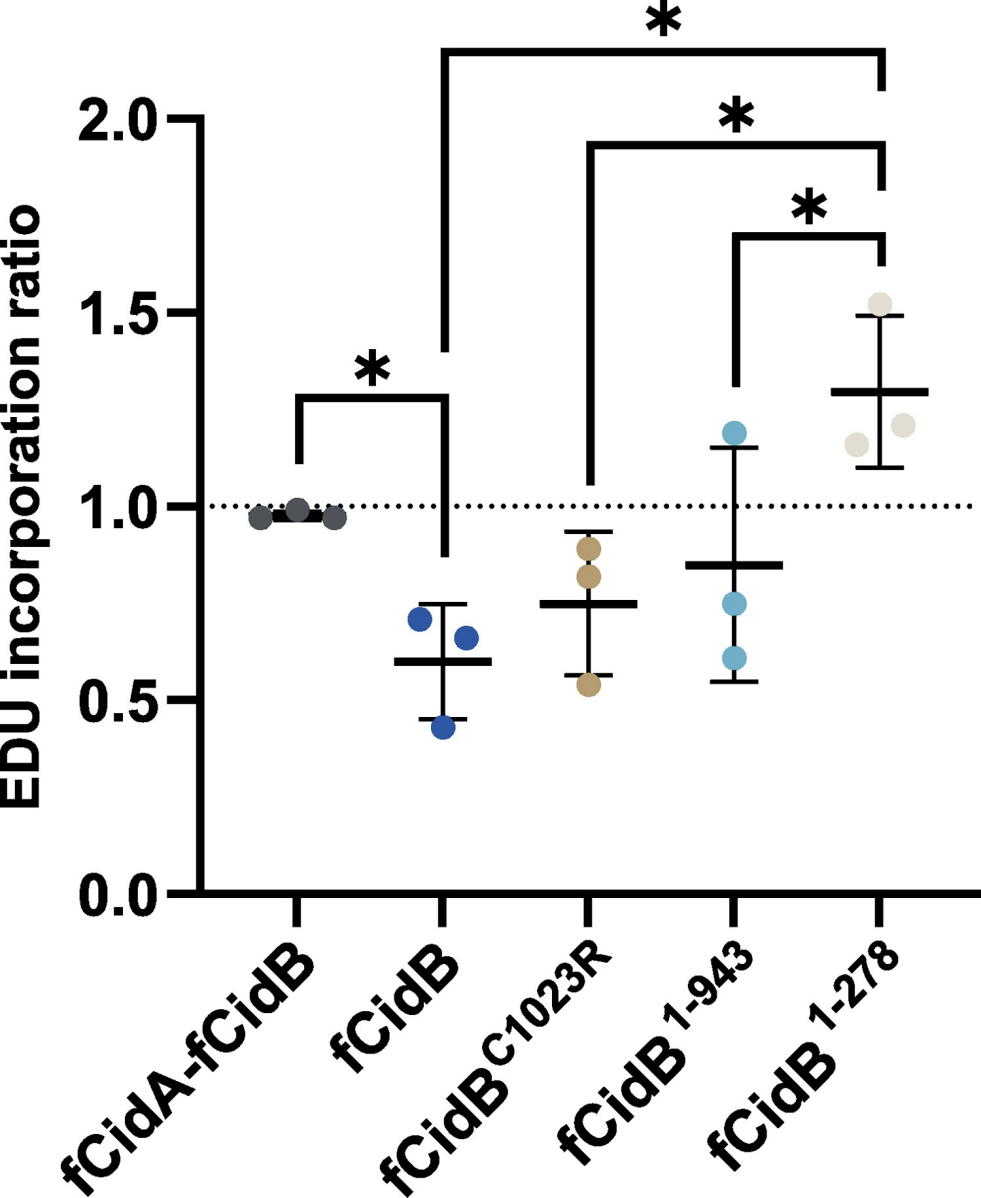
CidB can perturb S-phase progression without a catalytically active DUB domain. FACS analyses of EdU incorporation levels in transfected cells over non-transfected S2R+ cells. The analysis compiles three independent experiments, middle bar is mean, error bars are SD. The * indicates a p < 0.05 with a one-way ANOVA followed by multiple comparisons.

Previous studies suggested that DNA replication defects occur in CI, as the replication protein PCNA accumulates on paternal chromosomes in natural or transgenic CI [11,22]. Furthermore, we recently established that paternally-transmitted CidB^wPip^ forms nuclear foci that colocalize with PCNA as well as with the replication stress marker RPA on paternal chromatin during the first zygotic mitosis [11]. To explore to what extent CidB prevents DNA replication during S-phase and to establish which protein domains are involved, we carried out thymidine analog EdU incorporation experiments in the presence of either wild-type CidB or mutants. S2R+ cells were incubated for 4 hours with EdU, then fixed to reveal the incorporated EdU and to identify transfected cells based on the detection of the GFP signal associated with the expression of CidB full length or mutants (see Methods). The level of EdU incorporation in transfected cells was compared by FACS analyses to those obtained in their non-transfected counterparts and expressed as a ratio (Fig 7). S-phase progression in presence of fCidB with fCidA is undistinguishable from non-transfected cells and leads to a ratio of 1. In contrast, expression of fCidB alone reduces EdU incorporation levels, as does the expression of the DUB mutants fCidB^**C1023R**^ and fCidB^**1-943**^ although in a slightly milder manner. Expression of CidB CAD with fCidB^**1-278**^ does not perturb S-phase progression unlike the DUB mutants. Together, this dataset indicates that CidB poisons S-phase progression, independently of the DUB domain, and this defect is likely to be the cause of S2R+ cell death.

## Discussion

### Cid effectors are chromatin interactors

The individual expression of Cid effectors in the heterologous S2R+ cell system revealed that most CidA is efficiently exported out of the nucleus to remain cytoplasmic. In contrast, CidB alone is localized in the nucleus in interphase and intriguingly accumulates around the nucleolus. The relevance of perinucleolar CidB foci to CI is unclear, but may indicate a preference for a specific chromatin organization enriched with CidB interactor-s-. After NEB, CidA as well as a series of loss-of-function CidB mutants expressed separately decorate the mitotic chromatin evenly. The chromatin associated domains we identified, contained in the N- terminus region of CidB and C-terminus part of CidA, of about 278 and 52 residues respectively, do not give insights on the molecular nature of their interactors. CidB CAD has a nuclease fold domain which is not sufficient *per se* to decorate the condensed chromatin and a STE transcription factor-like domain previously identified [19] constitutes the CidA CAD. A CidA mutant deleted of its STE sequesters its cognate partner in the cytoplasm during mitosis, indicating that this domain is dispensable to neutralize CidB, and in agreement with data from a mutagenesis on Cid^**wMel**^ effectors [20]. It also strongly suggests that wild-type CidA-CidB complexes are anchored to the chromatin by the STE domain. This CidA driven localization of CidB may help to stabilize the toxin in the male germline nuclei until CidA eviction. During CidA removal in postmeiotic spermatids, CidB possibly relocates to another chromatin target thanks to its own CAD. Although a mutant CidB was previously show to weakly bind DNA, whether CidB toxicity requires a direct interaction with DNA is currently unknown [23]. Importantly, CidB loss-of-function mutants endowed with an intact CAD as well as the CAD expressed alone associate with the chromatin without causing any detectable defects in S2R+ cells or in transgenic *D*. *melanogaster* -i.e. CidB^**ΔNuf3**^-. This demonstrates that the CAD interaction with the host chromatin is not sufficient to drive toxicity but the role of the CAD docking CidB onto the chromatin is nonetheless essential for the toxin to prevent S-phase completion.

### CidA-CidB mechanisms of interaction and functional consequences

We established the minimum interface regions required on CidA to interact head-to-tail with CidB interface regions. CidA interface I is essential to ensure binding to CidB unlike interface III that appears dispensable. This last result differs from a previous study [13] that analyzed CidA interface regions by pull-down experiments and yeast cell growth assays and found subsets of interface III to be essential. This difference may arise from our partial deletion of interface III or because the CidA variants analyzed are different. Similarly, we established that CidB interface II together with interface III are sufficient to bind to a neutralizing CidA. When all interfaces are present, CidA prevents CidB direct chromatin binding and toxicity, to eventually anchor its complex with CidB to the chromatin through its STE-like domain. This suggests an affinity competition between CidA and the cellular partners of CidB whose interactions sites are not accessible when the antitoxin is present. Importantly, CidA prevents CidB CAD to interact with its target(s), even in absence of an intact interface III in CidA^**1-325**^. Because the corresponding interface III on CidB is located in its CAD, it is possible that CidA binding causes a local steric clutter that blocks any CidB interaction with the chromatin. CidA binding also neutralizes the CidB 278–943 region essential to CidB toxicity. Contacts between interface residues in a CidA-CidB complex are dependent on their 3D structure and can be perturbed by deletions outside the interface regions, affecting i.e. the protein folding. Hence other protein regions appear essential to modulate the affinity between partners. For instance, the Nuf1 domain is important for CidB to maintain a greater affinity for CidA over its chromatin partner. When kept away from the chromatin by a membrane anchorage, the CidB^**ΔNuf1**^- CidA^**CAAX**^ partners maintain their association. However, when the complex can interact with the chromatin at NEB, i.e. CidB^**ΔNuf1**^-CidA, CidA preferentially associates with its chromatin partner in an exclusive manner and the complex is disrupted. Deletion of the Nuf4 domain located outside interface regions allows CidB to relocate a CidA STE deletion mutant to the chromatin, while this STE mutant normally prevents a wild-type CidB to interact with the chromatin. Hence the CidA-CidB interaction is likely to rely on both interface regions as well as other Nuf domains to ensure to CidB a binding affinity superior to those of its cellular targets. Of note, it is important to keep in mind that the modulation of affinity between these partners obtained by deletion mutagenesis methods can arise from the loss of a given sequence but also from induced protein folding defects.

An antitoxin function of CidA achieved by masking crucial CidB interaction domains with its cellular targets is in agreement with a proteomic study showing that CidA scrambles an interacting proteome of CidB [24]. *In cellulo*, CidA blocks both CidB nuclear import and its direct binding to the chromatin, but nonetheless anchors the complex on the chromatin after NEB. Of note, CidB nuclear import occurs with the CAD-containing mutant CidB^**1-278**^ but is prevented with i.e. CidB^**ΔNuf2**^, CidB^**ΔNuf3**^ and CidB^**ΔNuf4**^ deletion mutants which nonetheless allow an interaction with the chromatin through the CAD after NEB. This indicates that the same N-terminal region of CidB is involved in both a nuclear import function and a chromatin interaction, possibly through interactions with different host targets.

### CidA protects and escorts CidB in the nucleus

The neutralization of CidB during spermatogenesis, its stability and proper loading in sperm nuclei are of paramount importance to cause CI after fertilization. Because CidA-CidB complexes can access the chromatin after NEB and anchor it thanks to the CidA STE domain, CidA appears sufficient to provide an access to CidB into the nucleus of mitotic and meiotic male germ cells. A cell-cycle dependent shuffling of CidA-CidB complexes between the nuclear and cytoplasmic compartments occurs *in cellulo*. We think that the same mechanism happens in early spermatogenesis since both localizations are observed [11]. It suggests that nuclear export mechanisms can detach the STE-linked CidA-CidB complexes from the chromatin, unlike CidB alone, until after meiosis where both proteins are found in the nuclei of differentiating spermatids.

A loss-of-function CidB mutant able to bind to the chromatin and endowed with its DUB domain (UAS-tCidB^**ΔNuf3**^) expressed alone or in combination with a wild-type CidA (UAS-tCidA-tCidB^**ΔNuf3**^) allowed us to explore the influence of the antitoxin on CidB dynamics *in vivo*. Only a co-expression with its CidA partner maintains fluorescence levels of this Cid mutant comparable to the wild-type CidA-CidB complex demonstrating that the antitoxin is essential to stabilize CidB during spermatogenesis. Once CidA is degraded during the histone-to-protamine transition, the toxin might be stabilized through its interaction with new host targets.

### The DUB domain catalytic activity participates to CI by shielding CidB against protein degradation

One currently debated question regarding the mechanism of CI is the role of CidB deubiquitylase activity in driving toxicity [25]. Transgenic co-expression of CidA with CidB in *D*. *melanogaster* males was initially reported to elicit a robust CI in eggs, abolished with a CidB DUB mutant whose catalytic activity is dead. The same study established that CidA binding to the toxin does not prevent the *in vitro* cleavage of K48 and K63-linked ubiquitin chains, suggesting that a role for CidA might be to sequester CidB away from its DUB substrate-s- [8]. We previously showed that transgenic males bearing the CidB DUB mutated transgene UAS-tCidA-tCidB^**C1025A**^ do trigger CI when induced by a Gal4 driver, suggesting that CI is not a direct consequence of a CidB substrate deubiquitylation in the paternal chromatin [11]. Additional lines of evidence support here an indirect role of the DUB activity in CI. First, S2R+ cell death was obtained with mutants whose DUB domain was either mutated to prevent catalytic activity and minimize ubiquitin binding (CidB^**C1023R**^) or deleted (CidB^**1-943**^). Second, CidA-CidB complexes can remain in the nucleus during interphase without causing apoptosis in S2R+ cells, thus contradicting the hypothesis that CidA relocates the CidB deubiquitylase -i.e. away form the nucleus- in order to neutralize its toxic activity. Finally, we created a series of neutralizing deletions mutants *in cellulo* (i.e. fCidB^**ΔNuf2**^, fCidB^**ΔNuf3**^, fCidB^**ΔNuf4**^) and in *D*. *melanogaster* transgenics (tCidB^**ΔNuf3**^) which all reside in the proper chromatin environment necessary to elicit toxicity, thanks to the N-terminus CAD. Despite the presence of an active DUB in all these mutants, as demonstrated with deubiquitin cleavage assays using a CidB^**762-1143**^ mutant containing the DUB domain [8], they do not elicit cell death or CI.

The DUB deletion in UAS-tCidA-tCidB^**1-943**^ leads to a rapid loss of detectable CidB mutant and associated CidA in early spermatogenesis and, accordingly, males expressing tCidB^**1-943**^ do not induce CI. In S2R+ cells as well, the fluorescence level of fCidB^**1-943**^ becomes reduced in a majority of cells during time-lapse experiments, suggesting that the loss of the DUB domain may render the CidB mutant protein intrinsically unstable independently of the loss of DUB catalytic activity. Our previous analyses reported that the level of CidB expression in transgenic testes depends nonetheless on its DUB domain activity, since UAS-tCidA-tCidB^**C1025A**^ dramatically reduces the level of CidB expression in the germline of males nonetheless able to induce CI [11]. Unlike tCidB^**1-943**^, tCidB^**C1025A**^ remains expressed with tCidA in early male germ cells as with wild-type tCidB does. We postulate that a major function of the DUB domain is to protect CidB when no longer escorted by CidA, in a perhaps cis or trans-acting manner against detrimental K63 or K48 polyubiquitylation mechanisms leading otherwise to proteasomal degradation of the toxin.

### Towards a molecular mechanism for CidB toxicity

This new role of the DUB domain invites us to revisit what confers CidB toxicity in CidB-induced cell death and CI. Three different regions appear necessary in CidB to induce CI, limited to a set of two regions to trigger cell death *in cellulo*. First, a CAD ensures its anchoring to the proper microenvironment, which by itself is harmless for the host cell. Although no *bona fide* DNA binding domain has been identified in CidB, this possibility cannot be excluded, yet an interaction with dsDNA only would not explain a perinucleolar accumulation in S2R+ cells unless this specific localization required the binding of additional targets. Its presence in both condensed and uncondensed chromatin all along the cell cycle indicates however that its binding partner is likely to be constitutive of the chromatin and does not vary according to the cell cycle. Second, a region spanning from a.a. 278 to 943 drives CidB toxicity when associated to the CAD. Third, the DUB domain catalytic activity appears essential to protect CidB from degradation but is dispensable to drive toxicity *in cellulo*.

Upon CidB expression *in cellulo*, S-phase can still largely progress but stops before completion, suggesting that a late phase of DNA replication may be affected. We think that CidB-induced S phase defects are likely to activate a DNA replication checkpoint *in cellulo*, but not *in vivo* in the male pronucleus, resulting in different terminal phenotypes. Our characterization of *w*Pip CidB functional regions has now paved the way to discover their respective host cell interactors to elucidate the mechanism of toxicity in S-phase leading to Cytoplasmic Incompatibility.

## Materials and methods

### *Drosophila* cell culture, transfection and imaging

*D*. *melanogaster* S2R+ cell lines S2R+ cells were obtained from the *Drosophila* Genomics Resource Center (DGRC) and cultured in Schneider’s *Drosophila* medium (Dutscher #L0207-500) supplemented with 10% Fetal Bovine Serum (Dutscher #S1810-500) at 25°C.

For live microscopy, cells were plated in 35 mm glass bottom dishes (Cellvis #D35-20-1.5-N) or 24-wells plates (Greiner Bio One Sensoplate) and transfected with Lipofectamine 3000 (Invitrogen #L3000008) with 500ng of purified plasmid DNA, according to manufacturer’s instructions. Transfected cells were observed between 24 and 48 hours after transfection either directly by confocal microscopy or in time- lapse experiments. More than 100 transfected cells were observed in at least three independent experiments for each condition. Interphasic localizations of Cid effectors illustrated in Figs 3 to 5 are representative of the 1^st^ interphase post-transfection. Confocal imaging was performed either with a Leica SP5-SMD microscope equipped with a 63X 1.4 NA objective lens, a Dragonfly spinning disk with a 60X Plan Apo lambda 1.4 NA objective lens or a Zeiss LSM980 equipped with a 63X Plan Apo oil 1.4NA objective lens. In some cases, cells were treated 3hrs with 10μM colchicine (Sigma, #C9754) prior to imaging to facilitate live imaging of mitotic events. Time-lapse experiments were performed in three independent transfection experiments, and images were acquired every 20 min for 48hrs with an inverted Nikon Ti2 widefield fluorescent microscope with a 20X Plan Fluor 0.75NA oil objective lens and a Prime 95B back-illuminated sCMOS camera. Cell death and mitotis events were manually scored (S2 Fig).

### Plasmid constructs for fluorescent Cids expression in *D*. *melanogaster* cells

A synthetic cassette (Genscript) containing the mkate2-T2A-sfGFP bloc was inserted in the Multiple Cloning Site of a *Drosophila* cell vector based on the pMT-V5-HisC (Invitrogen #V412020) modified to have an Actin5C promoter. The CidA_IV(δ/1) and CidB_IV(a/2) genes were synthesized after codon optimization for expression in *D*. *melanogaster* cells (Genscript). The third 73 bp intron of the *D*. *melanogaster nanos (nos)* gene was inserted close to the 5’ end of CidB to avoid toxic leak encountered in *E*. *coli* [11]. All plasmids were obtained by Gibson cloning using the NEBuilder Hifi DNA Assembly kit (NEB #E5520S) and verified by Sanger sequencing. Deletion mutants were created by PCR amplification with the Q5 polymerase (NEB #M0491S).

### Flow cytometry analyses

Cell viability assays were conducted as previously described [11]. Briefly, cells were analysed in three biological replicates at 2 and 4 days post-transfection and a growth rate calculated according to the following formula:

Log2 (x at Day 4 / x at Day 2)

where x is the proportion of fluorescent cells at the given time-point. A fold change equal or superior to 0 is observed when transfected cells grow at similar rate compared to non-transfected cells. In contrast, a negative fold change reflects a slower growth or cell death between day 2 and 4. Data were acquired with a Novocyte ACEA cytometer and analyzed with the NovoExpress (ACEA) software. Statistical analyses are based on an ordinary one-way ANOVA test followed by multiple comparisons: mKate2-sfGFP vs fCidB: P<0.0001, n = 3.

### EdU incorporation assays and Immunofluorescence stainings

S2R+ cells were plated on 12-well plates and transfected as described above. After 24h, 10μM EdU was added to the cell culture medium media for 4h. Next, cells were mechanically detached and transferred into Eppendorf Protein LoBind tubes (Merck #EP0030108116), rinsed in PBS Tween 0.1%—by centrifugation at 2,000 rpm for 1 min- before a 10 min fixation in 3.2% paraformaldehyde. After two washes in PBS Tween 0.1% cells were incubated with an anti-GFP rabbit antibody (1/1000) overnight (Genscript #A01704), rinsed three times and incubated for 2 hours with a fluorescent goat anti-rabbit IgG antibody, FITC conjugate (Chemicon #AP132F). After two washes, EdU staining was immediately performed according to manufacturer’s instructions (ThermoFischer Click-i Plus EdU Alexa Fluo 647 Flow Cytometry Assay Kit, C10634). Cells were analyzed by flow cytometry on a sampling of 200,000 counted events. The EdU incorporation ratio was obtained with the formula: x transfected GFP-positive cells / x untransfected GFP-negative cells where x is the mean EdU signal. This experiment has been performed with three biological replicates. Statistical analyses are based on an ordinary one-way ANOVA test followed by multiple comparisons. fCidA-fCidB vs. fCidB: P = 0.038, fCidB vs. fCidB^**1-278**^: P = 0.0013, fCidB^**C1023R**^ vs. fCidB^**1-278**^: P = 0.006, fCidB^**1-943**^ vs. fCidB^**1-278**^: P = 0.0177, n = 3.

### Drosophila stocks

Flies were reared at 25°C on a classical agar, yeast, corn flour fly medium.

All transgenic stocks were inserted in the *PBac{y[+]-attP-9A}VK00027* platform on chromosome 3R (89E11) by PhiC31-mediated germline transformation [26]. The *y w; pUASP-6His-V5-CidA-T2A-Flag-CidB-attP/+* stock expressing wild-type effectors was previously described [8]. *Sevelin* is a wild-type *D*. *melanogaster* stock. The *bam-Gal4* driver is a third chromosome insertion of *P{w[+mC] = bam-GAL4*:*VP16}* [27]. All stocks were checked for the absence of *Wolbachia* infection by fluorescent microscopy and PCR detection of 16S rRNA [28].

### Drosophila fertility tests

Adult virgin females carrying the *w*Pip effector transgenes were crossed with *bam*-Gal4 driver males and F1 adult males carrying both *white*+ marked transgenes were selected based on darker eye pigmentation.

To measure fertility, fifteen 0 to 48-hour-old virgin wild-type Sevelin females were aged for 2 additional days at 25°C in presence of fifteen 2 to 4-day-old males of the genotype of interest. Females were then allowed to lay eggs on grape-juice agar plates for 12 hours. Embryos were counted and then let to develop for at least 36 hours at 25°C. Unhatched embryos were counted over four consecutive days to determine hatching rates.

### Immunofluorescence and imaging of Drosophila testes

Testes from 2 to 4-day-old males were dissected in PBS-T (PBS 1X with 0.15% Triton X-100) and fixed for 20 min in 4% PFA at room temperature. Testes were washed 3 times in PBS-T and incubated with primary antibody overnight at 4°C. After three 20 minutes washes in PBS-T, they were incubated with secondary antibodies at room temperature for 2 hours. Testes were then mounted in Dako mounting medium (Agilent, #S3023) containing 1 μg/ml DAPI.

Primary antibodies used were mouse monoclonal anti-V5 (Invitrogen #R960-25, 1:500), mouse monoclonal anti-Flag (clone M2—Sigma-Aldrich #F3165, 1:1000) and rabbit polyclonal anti-acetyl Histone H4 (Merck Millipore #06–589; 1:500). Secondary antibodies were used at a 1:1000 dilution and included: Alexa Fluor 647 goat anti-rabbit IgG (Invitrogen, # AB-2535813), Alexa Fluor 555 goat anti-mouse IgG1 (Invitrogen #AB-2535769) and Alexa Fluor 488 goat anti-mouse IgG2a (Invitrogen #AB-2535771). Images were acquired on an LSM 800 confocal microscope equipped with a 40X 1.4 NA objective lens (Carl Zeiss). Images were processed with Image J and GIMP.

## Supporting information

S1 Fig Blast comparison between the CidA and CidB variants from the *w*Pip(Pel) reference genome used in [14] and the variants used in cellulo in this study, CidA_IV(δ/1) and CidB_IV(a/2) [9]. Previously identified protein domains are represented on the schematic drawing and reported as colour-coded boxes on the protein sequences, and the interface regions I, II and III are shown in red.
(TIF)

S2 Fig Quantification of apoptosis and mitotic events in transfected cells by time-lapse microscopy. Percentages of mitotic and apoptotic events of transfected cells are compiled from three independent transfection experiments during 48hr-long time lapse recordings. The number of transfected cells examined is indicated to the right of each column.
(TIF)

S3 Fig Instability of some Cid mutants in S2R+ cells. Confocal images of cells expressing (A) fCidB^**1-737**^ forming aggregates unless fCidA is coexpressed and (B) an example of cell in which fCidB^**1-943**^ fluorescence disappears to become limited to a focus in the perinucleolar area. Scale bar = 10 μm.
(TIF)

S4 Fig Unstable CidA mutants. Confocal images of cells expressing fCidA^**1-200**^ or fCidA^**Δ1–100**^ forming fluorescent cytoplasmic aggregates. Scale bar = 10 μm.
(TIF)

S1 Movie Expression of CidB at low level also leads to cell death. Time lapse of two S2R+ cells expressing CidB at different levels of intensity. Arrows point towards the two nuclei. Images were recorded every 15 minutes.
(MP4)

S2 Movie Subnuclear localization of CidB. Animation through Z stacks of confocal images of cells transfected with fCidB partially colocalizing with the perinucleolar heterochromatin revealed with Hoechst (an example is given -arrows-).
(MP4)

S3 Movie CidA-CidB complexes can remain in the nucleus in interphase without preventing mitosis. Time lapse experiment over 24 hours showing two successive mitosis of a S2R+ cell expressing fCidA-fCidB.
(MP4)

S1 Table *Drosophila* embryo hatching rates. (DOCX)
