# Supplementary material for: Functional analysis of Wolbachia Cid effectors unravels cooperative interactions to target host chromatin during replication
Source: PLoS Pathog. 2023 Mar 16;19(3):e1011211. doi: 10.1371/journal.ppat.1011211 (PMC10047532; doi:10.1371/journal.ppat.1011211)
Supplement: S1 Table — (DOCX) [file ppat.1011211.s008.docx]

**S1 Table. *Drosophila* embryo hatching rates**

| Males | Females | Number of eggs | Hatching rate (%) |
| --- | --- | --- | --- |
| *WT* | *WT* | 938 | 89.02 |
| *bam-Gal4 > UAS-tCidA-tCidB* | *WT* | 638 | 0 |
| *bam-Gal4 > UAS-tCidB****^∆Nuf3^*** | *WT* | 775 | 86.58 |
| *bam-Gal4 > UAS-tCidA-tCidB****^∆Nuf3^*** | *WT* | 737 | 81.28 |
| *bam-Gal4 > UAS-tCidA-tCidB****^1-943^*** | *WT* | 704 | 90.63 |
|  |  |  |  |
| *+ > UAS-tCidB****^∆Nuf3^*** | *WT* | 781 | 85.66 |
| *+ > UAS-tCidA-tCidB****^∆Nuf3^*** | *WT* | 869 | 80.67 |
| *+ > UAS-tCidA-tCidB****^1-943^*** | *WT* | 856 | 94.86 |
